# Tropical legume grass forage (*Stylosanthes hamata* L.) reduces enteric methane yield and intensity and improves milk yield in Fulani zebu suckler cows

**DOI:** 10.1016/j.vas.2026.100784

**Published:** 2026-07-24

**Authors:** G.X. Gbenou, B.M. Orounladji, B. Adama, F. Sanou, D. Bastianelli, O. Sib, S. Sanogo, L. Bonnal, E. Vall, L.H. Dossa, M.H. Assouma

**Affiliations:** a Laboratoire des Sciences Animales, Faculté des Sciences Agronomiques, Université d’Abomey-Calavi, 526 Cotonou, Benin; b SELMET, University of Montpellier, CIRAD, INRAE, Institut Agro, Montpellier, France; c Centre International de Recherche Développement sur l’Elevage en zone Subhumide, 454 Bobo-Dioulasso, Burkina Faso; d CIRAD, UMR SELMET, Dakar, Senegal; e CIRAD, UMR SELMET, F-34398 Montpellier, France; f CIRAD, UMR SELMET, Nairobi, Kenya

**Keywords:** Methane mitigation, Productivity, Suckler cow, Protein supplementation, Sub-Saharan Africa

## Abstract

In sub-Saharan Africa, enteric methane (eCH_4_) emissions represent the principal source of greenhouse gases from ruminant livestock. Most milk in this region is produced by suckler cows of local breeds, yet very few references with *in vivo* eCH_4_ measurements are available. This study assessed the impact of protein supplementation with *Stylosanthes hamata* L. on eCH_4_ emissions in Sudanese Fulani zebu suckler cows. The experiment was conducted under stable conditions with 10 animals: five steers (38 months old, 179 ± 20.3 kg body weight - BW) and five suckler cows (75 months old, 204 ± 13.3 kg BW). Two dietary treatments were offered: a control diet of *Brachiaria ruziziensis* (100%; hereafter Brach), and an experimental diet combining *B. ruziziensis* with *S. hamata* in a 75:25 ratio (hereafter Brach+Stylo). Each diet was provided at 2.5% BW (dry matter [DM] basis) per animal for 4 weeks (2 weeks of adaptation and 2 weeks of data collection). Intake, milk production and gas emissions (GreenFeed® system) were measured (mean ± SD) in cows, while apparent digestibility was measured in steers. Daily DM intake was 17.2 ± 1.66 g/kg BW for Brach and 20.0 ± 3.76 g/kg BW for Brach+Stylo. DM digestibility was 0.46 ± 0.05 for Brach and 0.50 ± 0.06 for Brach+Stylo, while daily milk yield was 1.4 ± 0.08 kg and 2.0 ± 0.08 kg, respectively. The eCH_4_ yields were 28.4 ± 4.50 (g/kg DMI) for Brach and 24.4 ± 3.90 (g/kg DMI) for Brach+Stylo corresponding to 72.2 ± 9.51 (g/kg milk) and 50.4 ± 4.83 (g/kg milk), respectively (*p* < 0.05). Our results indicate that protein supplementation with *S. hamata* increases DM intake, DM digestibility, and milk yield, while reducing eCH_4_ yield and intensity. These findings highlight the potential of targeted protein supplementation to improve productivity and mitigate eCH_4_ emissions in suckler cow–based dairy systems.

## Highlights

• *Stylosanthes spp*. is among the most widely used tropical livestock forages.
• Stylosanthes supplementation improve intake, digestibility, and milk production.
• Methane yield and intensity decreased with tropical legume supplementation.
• Locally adapted feeding strategies can help produce more with lower environmental impact.

## Graphical abstract

**Figure:**
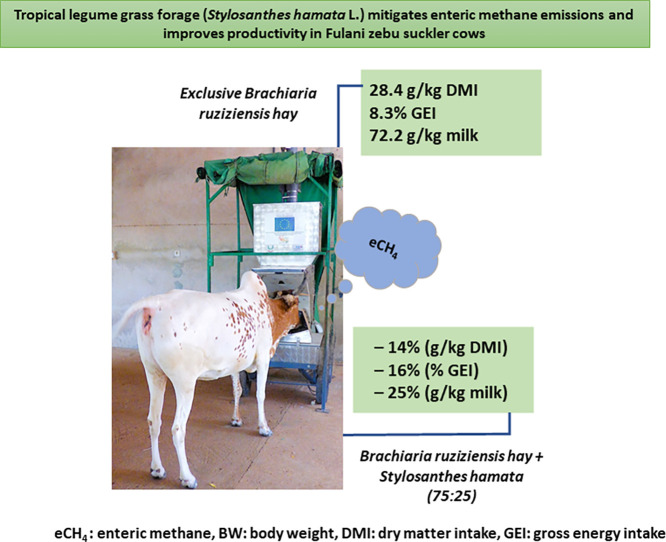

## 1 Introduction

Across sub-Saharan Africa (SSA), and particularly in the West African region, population expansion and accelerating urbanisation are driving a growing demand for animal-source foods, placing increasing pressure on livestock farmers to intensify production (WBG, 2023). This intensification of livestock farming raises major environmental concerns, particularly with regard to the greenhouse gas (GHG) emissions associated with animal production systems. Among these GHGs, enteric methane (eCH_4_), produced by microbial fermentation in the rumen of ruminants, is a major source of emissions. It accounts for around 60% of total CO_2_ emissions from livestock systems (De Figueiredo et al., 2017), a proportion likely to increase as livestock farming expands.

Suckler cow breeding, which plays a key role in food security and the rural economy, across SSA, and particularly in the West African region, faces significant feed constraints. A major challenge is the low quality of tropical forages during the dry season (Hiernaux & Assouma, 2020). These forages, often deficient in protein and energy, result in less efficient digestion and higher eCH₄ emissions per unit of intake or production (Assouma et al., 2019). Enteric methane represents a loss of 2 - 12% of gross energy intake (GEI) in ruminants, underscoring both its environmental and nutritional implications (IPCC, 2019). It is therefore crucial to identify locally adapted feeding strategies that enhance livestock productivity while reducing their environmental footprint, thereby fostering more climate-resilient and sustainable livestock systems.

Incorporating legume forages into ruminant diets represents a promising strategy for mitigating eCH₄ emissions (Abraham et al., 2023; Archimède et al., 2011). These protein-rich plants, which contain secondary compounds such as mimosine, tannins, saponins and flavonoids (Archimède et al., 2011), can alter the composition of the ruminal microbiota, and have been shown to decrease eCH₄ emissions *in vivo* (Archimède et al., 2011). *Stylosanthes spp*., a perennial legume, is among the most widely used forages in cultivated pastures and is increasingly introduced into dairy and non-dairy livestock feed under fresh, hay, silage and pellets form through the world (Da Silva et al., 2022; Hall et al., 2004; Paciullo et al., 2014; Schultze-Kraft et al., 2023), particularly in the West African region (Ouédraogo et al., 2023). However, despite increasing interest in tropical legumes as methane-mitigating feeds, *in vivo* evidence on the effects of *Stylosanthes hamata* supplementation on eCH_4_ emissions remains limited, particularly in African cattle breeds. Its adaptability to tropical environments, ability to improve forage quality, and suitability for low-input production systems position *S. hamata* as a promising climate-smart feed for smallholder livestock systems in SSA. As the dairy sector expands, and the need for climate-smart feeding strategies increases, evaluating the potential of this locally available forage resource becomes essential. This study therefore investigated intake, digestibility, milk production, and eCH_4_ emissions in Sudanese Fulani zebu suckler cows supplemented with *S. hamata*.

## 2 Materials and methods

Ethical approval for this study was obtained from the CIRDES (Centre International de Recherche-Développement sur l’Élevage en zone Subhumide) Ethics Committee for Animal Experiments on 15 April 2021 (Application No 006/Mars/2021/CE-CIRDES). All procedures involving animals were conducted in strict accordance with institutional guidelines for the care and use of animals in research.

### 2.1 Experimental site

The experiment was conducted at the CIRDES experimental station (ventilated barn: 25 m × 10 m) in Bobo-Dioulasso (11°10′37′' N, 4°17′52′' W), western Burkina Faso (Gbenou et al., 2024). The area is characterised by a tropical subhumid climate with three seasons: a wet season (May-October), a cool dry season (November-February), and a hot dry season (March-May). The study was conducted during the wet season, when mean temperature and rainfall were 26 ± 0.5 °C and 691 mm, respectively.

### 2.2 Animals, feed, and experimental design

Ten Sudanese Fulani zebu cattle (five steers and five suckler cows) were included in the study. The steers were 38 months old, with an average body weight of 179 ± 20.3 kg (equivalent to 0.72 Tropical Livestock Units, TLU). The suckler cows were 75 months old and had an initial body weight (BW) of 204 ± 13.3 kg (0.82 TLU). All animals were housed individually in 9 m^2^ pens within an experimental barn.

Two forages species were evaluated: the grass *Brachiaria ruziziensis* (Brach) and the herbaceous legume *Stylosanthes hamata* L. (Stylo), harvested at maturity after 25 and 18 weeks, respectively. The forages were cultivated at the *Institut National de l’Environnement et des Recherches Agricoles* (INERA, Farako Bâ, Burkina Faso) on soils characterised by the following particle size distribution: clay (< 2 μm), silt (2–50 μm), and sand (50–2000 μm). Animals received half of their daily ration at 08:30 and the remainder at 16:30. Drinking water and lick stones were provided *ad libitum*.

The animals were assigned to two dietary treatments: Brach alone (100%) and Brach+Stylo (75:25) in a crossover design, allowing each animal to receive both dietary treatments and serve as its own control, thereby minimizing between animal variability and increasing the precision of treatment comparisons. The 8-week trial comprised two 4 week periods: 2 weeks of adaptation and 2 weeks of data collection. Animals received Brach+Stylo in the first period and Brach alone in the second. Each diet was offered to both steers and suckler cows at 2.5% of BW on a dry matter (DM) basis per animal. Measurements included body weight, feed intake, nutrient digestibility, milk yield, and gas emissions.

### 2.3 Feed intake and digestibility assessment

Body weight was recorded at the beginning and end of each week throughout the data-collection period. Voluntary feed intake was measured daily by weighing the feed offered and refused for each animal. Representative samples of both offered and refused diets (300 g per sample) were collected and stored individually. During each 14-day period, one offered sample was collected daily, yielding 14 samples per diet. Refusals were collected daily from each of five animals, resulting in 70 samples per diet. Actual intake composition was calculated from the chemical analysis of offered feed and refusals..

As described by Gbenou et al. (2024), each steer was fitted with a faecal collection bag, to which it was accustomed during the adaptation phase. Total faecal collection enabled the calculation of apparent digestibility for each diet (Eq. (1)). Digestibility was assessed in steers, as total faecal collection with faecal bags was impractical in suckler cows nursing calves under the experimental conditions. Each day, an 800 g representative sample of faeces was collected from each animal and stored separately. All samples were oven-dried immediately at 55 °C for 72 h and ground to 1 mm (SM 100 mill, Retsch GmbH, Haan, Germany) prior to chemical analysis.

(1) $$\begin{array}{c} \text{Apparent}\;\text{DM}\;\text{digestibility}=\left(\text{DM}\;\text{intake}-\text{DM}\;\text{excreted}\right)\;/\;\text{DM}\;\text{intake} \end{array}$$

### 2.4 Milk production measurement

Milk yield in suckler cows was recorded each morning at 06:00 daily during the 14-day data collection period. Calves were allowed to suckle twice daily (09:00 and 18:00), consistent with common farming practice. The milk consumed by calves was quantified by weighing them before and after each suckling using a scale, with intake calculated as the difference between the two weights. The total milk consumed per day was obtained by summing the intake from both suckling sessions and adding the weight of milk collected from each cow at 06:00. No correction was applied for residual milk remaining in the udder after suckling. Milk yield was expressed relative to DM intake according Eq. (2):

(2) $$\begin{array}{c} \text{Milk}\;\text{yield}\;\left(g/\text{kg}\;\text{DM}\;\text{intake}\right)=\text{Milk}\;\left(g/\text{day}\right)/\;\text{DM}\;\text{intake}\;\left(\text{kg}/\text{day}\right) \end{array}$$

#### 2.4.1 Enteric methane and carbon dioxide emission measurements

Methane and carbon dioxide emissions in cows were measured using the GreenFeed® system (GF - ID: 252, C-Lock Inc., SD, USA). During each animal visit, the instrument dispensed a 34 g pellet of bait every minute, up to a maximum of 0.51 kg per animal per day. The bait consisted of a 90:10 mixture of rangeland fodder and molasses, formulated to minimize interference with voluntary intake, digestibility, or methane emissions. Prior to mixing with molasses, the fodder was ground to pass through a 1 mm sieve, after which the mixture was pelleted to 8 mm diameter. Refused bait was collected and weighed for each animal at every GF visit, allowing calculation of the amount ingested and its inclusion in total daily intake.

Animals visited the GF at 06:30 am (following an overnight fast), 10:00 am (immediately post-feeding), 2:00 pm (during rumination), 6:00 pm (after feeding and at sunset), 9:00 pm (night), 12:00 am, and 3:00 am (rest period), reflecting their natural feeding behaviour. Each animal completed 98 GF visits per diet, exceeding the 20 and 29 visits reported by Manafiazar et al. (2017) and Gbenou et al. (2024), respectively. The mean duration per visit was 3 min 05 s ± 19 s with a minimum of 2 min 01 s and a maximum of 5 min 26 s.

At 04:00 am each day, when no animals were present, the GF was automatically calibrated for each gas using a certified gas mixture (CH_4_: 0.509 ppmv, CO_2_: 4.993 ppmv, H_2_: 0.010 ppmv, O_2_: 0.021 ppmv; Liquide Air, C-Lock Inc., USA). A CO_2_ recovery test was performed twice weekly, and filters were replaced when airflow dropped below 27 L/s. During the study, recovery rate, wind direction, and airflow averaged 98.6 ± 1.08% (Min = 92.78, Max = 99.56), 121.3 ± 28.57° (Min = 0.68, Max = 355.67), and 33.20 ± 3.01 L/s (Min = 21.76, Max = 38.16), respectively.

### 2.5 Chemical analysis of feed and feces

As described by Gbenou et al. (2024), the chemical composition of offered and refused forages, as well as of faeces, was determined using near-infrared spectroscopy (NIRS) at CIRAD (Baillarguet, France). For forages, the parameters analysed included ash, crude protein (CP), neutral detergent fibre (NDF), acid detergent fibre (ADF), acid detergent lignin (ADL), gross energy (GE), and *in vitro* digestibilities of DM (IVDMd) and organic matter (IVOMd). For faeces, ash, nitrogen, NDF, ADF, ADL, and GE were determined. The chemical composition of the intake was calculated from the composition of offered and refused forages. Table 1 presents the chemical composition of the diets.

**Table 1:** Nutritional quality of offered diets to zebu cattle.

Diet	OM (g/kg DM)	CP (g/kg DM)	NDF (g/kg DM)	ADF (g/kg DM)	ADL (g/kg DM)	IVDMD	IVOMD	GE (MJ/kg DM)
Brach	944.9 ^B^	17.2 ^B^	756.8	450.9 ^A^	83.4	0.30 ^A^	0.26 ^A^	19.0
Brach+Stylo	954.5 ^A^	23.4 ^A^	750.0	439.1 ^B^	86.7	0.34 ^B^	0.30 ^B^	19.2
SEM	1.87	0.83	3.02	2.47	1.43	4.95	4.52	3.12
P-value	0.007	<0.001	0.084	0.015	0.264	<0.001	<0.001	0.125

OM: organic matter, DM: dry matter, CP: crude protein, NDF: neutral detergent fibre, ADF: acid detergent fibre, ADL: acid detergent lignin, IVDMD: *in vitro* dry matter digestibility, IVOMD: *in vitro* organic matter digestibility, GE: gross energy, MJ: megajoule.

Brach: *Brachiaria ruziziensis* hay harvested at maturity stage, Stylo: *Stylosanthes hamata* harvested at maturity stage; SEM = Standard Error of the Mean.

^A,B^ Values with different superscripts within the same column differ significantly at *p* < 0.05.

### 2.6 Gas measurement data handling

Raw data (n = 980) generated by the GF for each gas were uploaded to the C-Lock web platform. Records corresponding to visits lasting <2 min or characterised by inadequate head positioning (sensor signal < 800 raw units) were excluded, resulting in the removal of 58 measurements (5.9%) per gas. The remaining spot values of eCH_4_ and CO_2_ (g/d) per visit and per animal (n = 922 per gas) were retained and incorporated in the data file obtained from C-Lock.

Following the linear regression method outlined by Coppa et al. (2021), outlier data, representing 0.54% of the dataset, were eliminated for each gas, leaving 817 measurements per gas for further analysis. Individual daily mean values of eCH_4_ flow were calculated by averaging all visit-level data per animal and per day for each diet (Equation 4). Because three measurements were obtained during the daytime (10:00 am, 2:00 pm, 6:00 pm) and four at night (6:30 am, 9:00 pm, 12:00 am, 3:00 am), overall daily eCH_4_ emissions were weighted by attributing equal importance to each measurement (Equation 3).

The eCH_4_ yield was then calculated using Equation 4, and, based on the gross energy intake (GEI), the eCH_4_ conversion rate (Ym) was computed according to Equation 5 provided by the IPCC (2019).

(3) eCH_4_ (g/day) = 0.5*[(eCH_4_.10:00 + eCH_4_.14:00 + eCH_4_.18:00) / 3] + 0.5*[(eCH_4_.06:30 + eCH_4_.21:00 + eCH_4_.00:00 + eCH_4_.03:00) / 4]

with eCH_4_.i= average eCH_4_ emissions (g/day) for visits within period i.

(4) eCH_4_ (g/kg milk) = eCH_4_ (g/day) / milk (kg/day)

(5) Ym (% GEI) = [(eCH_4_ x 55.65/1000) / GEI] x 100

with eCH_4_ in g/day and GEI in MJ/day.

### 2.7 Data analysis

All statistical analyses were performed using R software (R Core Team, 2025). Normality of data distributions was assessed with the Shapiro-Wilk test, and homogeneity of variances between groups was evaluated using Levene’s test. When both assumptions were satisfied, mean intake, digestibility, milk production, and gas emissions between the two groups were compared using Student’s *t*-test for paired samples. When either assumption was violated, comparisons were made using the non-parametric Wilcoxon test. The same procedures were applied to compared intake and refusals. As all animals received diets in the same sequence, period and treatment effects were confounded and not analysed separately; period and carryover effects were therefore disregarded.

## 3 Results

### 3.1 Nutritional quality of offered diets

The nutritional quality of the diets differed significantly (Table 1). The Brach+Stylo diet contained 36% more CP than the Brach diet (*p* < 0.05). No significant differences were observed in NDF and ADL contents between the diets, but the control diet was richer in ADF than the supplemented ones (*p* < 0.05).

### 3.2 Diet intake, refusals, and digestibility

Dry matter intake (DMI), organic matter intake (OMI), crude protein intake (CPI), neutral detergent fibre intake (NDFI), acid detergent fibre intake (ADFI) (g/kg BW), and gross energy intake (GEI) (MJ/kg BW) were significantly higher for the Brach+Stylo diet compared with the Brach diet (*p* < 0.05; Table 2). The CP content was lower, and the NDF content higher in refusals compared with the intake of the experimental diets (*p* < 0.05; Table 3). Refusals represented 29% and 28% of the feed offered for Brach and Brach+Stylo, respectively. Digestibility was significantly higher for the Brach+Stylo diet (*p* < 0.05; Table 4) but the CP digestibility was negative for both diets. The average daily bait intake was 120 ± 10.2 g DM per animal for both diets.

**Table 2:** Daily intake by Fulani zebu suckler cows (including bait intake).

Item	Brach	Brach+Stylo	SEM	P-value
DMI (kg)	3.5 ^B^	4.2 ^A^	0.25	0.045
DMI (g/kg BW)	17.2 ^B^	20.0 ^A^	0.93	0.027
OMI (kg)	3.3	4.0	0.24	0.054
OMI (g/kg BW)	16.1 ^B^	19.0 ^A^	0.91	0.021
CPI (kg)	0.08 ^B^	0.11 ^A^	0.01	0.021
CPI (g/kg BW)	0.39 ^B^	0.51 ^A^	0.04	0.020
NDFI (kg)	2.6	3.1	0.19	0.057
NDFI (g/kg BW)	12.7 ^B^	15.0 ^A^	0.69	0.023
ADFI (kg)	1.5 ^B^	2.0 ^A^	0.11	0.019
ADFI (g/kg BW)	7.4 ^B^	9.6 ^A^	0.39	0.005
GEI (MJ)	67.4	80.9	4.81	0.055
GEI (MJ/kg BW)	0.33 ^B^	0.39 ^A^	0.02	0.024

Brach: *Brachiaria ruziziensis* hay harvested at maturity stage, Stylo: *Stylosanthes hamata* harvested at maturity stage; SEM = Standard Error of the Mean.

DMI: dry matter intake, BW: body weight, OMI: organic matter intake, CPI: crude protein intake, NDFI: neutral detergent fibre intake, ADFI: acid detergent fibre intake, GEI: gross energy intake.

^A,B^ Values with different superscripts within the same row differ significantly at *p* < 0.05.

**Table 3:** Chemical composition of intake and refusals in Fulani zebu suckler cows.

Item	Brach		SEM	P-value	Brach+Stylo		SEM	P-value
	Intake	Refusals			Intake	Refusals		
OM (g/kg DM)	939 ^B^	959 ^A^	3.4	0.010	952	959	1.5	0.099
CP (g/kg DM)	20 ^A^	12 ^B^	1.4	0.025	25 ^A^	20 ^B^	0.9	< 0.001
NDF (g/kg DM)	742 ^B^	788 ^A^	7.8	0.001	753 ^B^	782 ^A^	5.1	0.028
ADF (g/kg DM)	435 ^B^	486 ^A^	8.6	0.041	481 ^B^	509 ^A^	6.0	0.035
GE (MJ/kg DM)	19	19	0.0	0.134	19	19	0.0	0.122

Brach: *Brachiaria ruziziensis* hay harvested at maturity stage, Stylo: *Stylosanthes hamata* harvested at maturity stage; SEM = Standard Error of the Mean.

OM: organic matter, DM: dry matter, CP: crude protein, NDF: neutral detergent fibre, ADF: acid detergent fibre, IVDMD: *in vitro* dry matter digestibility, IVOMD: *in vitro* organic matter digestibility, GE: gross energy, MJ: mega joule.

^A,B^ Values with different superscripts within the same row differ significantly at *p* < 0.05.

**Table 4:** Digestibility of diets ingested by Fulani zebu suckler cows.

Digestibility	Brach	Brach+Stylo	SEM	P-value
DMd	0.46 ^B^	0.50 ^A^	0.009	0.003
OMd	0.48 ^B^	0.52 ^A^	0.011	0.013
CPd	−0.38 ^B^	−0.13 ^A^	0.048	< 0.001
NDFd	0.53 ^B^	0.60 ^A^	0.014	0.009
ADFd	0.52 ^B^	0.61 ^A^	0.017	0.004
GEd	0.47 ^B^	0.50 ^A^	0.008	0.022

Brach: *Brachiaria ruziziensis* hay harvested at maturity stage, Stylo: *Stylosanthes hamata* harvested at maturity stage; SEM = Standard Error of the Mean.

DMd: dry matter digestibility, OMd: organic matter digestibility, CPd: crude protein digestibility, NDFd: neutral detergent fibre digestibility, ADFd: acid detergent fibre digestibility, ADL: acid detergent lignin digestibility, GEd: gross energy digestibility.

^A^^,B^ Values with different superscripts within the same row differ significantly at *p* < 0.05.

### 3.3 Milk production

**Fig. 1:**
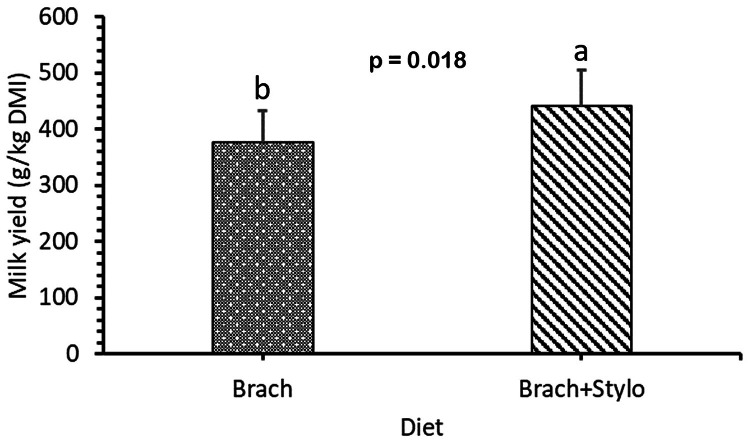
Milk yield in Sudanese Fulani zebu suckler cows.

Milk production differed significantly between treatments. Average daily milk production was 1.4 ± 0.09 for cows fed the Brach and 2.0 ± 0.08 kg for those fed the Brach+Stylo diet (*p* < 0.05). Milk yield (g/kg DMI) was 23% higher in the Brach+Stylo treatment compared with the Brach treatment (Fig. 1; *p* = 0.04).

### 3.4 Enteric emissions in suckler cows

Measured eCH_4_ emissions ranged from 89 to 116 g/d for both treatments, with no significant differences between diets (Table 5). With Brach+Stylo, eCH_4_ yield decreased from 28.4 to 24.4 g/kg DMI (-14%, *p* = 0.043) and from 30.3 to 25.6 g/kg OMI (-16%, *p* = 0.022). The eCH_4_ yield per unit of digestible DMI and OMI followed the same pattern, with significant differences (*p* < 0.05). The eCH_4_ intensity (g/kg milk) was reduced by 25% in the Brach+Stylo diet (*p* = 0.001).

**Table 5:** Enteric methane emissions in Fulani zebu suckler cows.

Item	Brach	Brach+Stylo	SEM	P-value
eCH_4_ (g/d)	98.0	99.8	2.58	0.753
eCH_4_ (g/kg BW)	0.48	0.48	0.013	0.886
eCH_4_ (g/kg DMI)	28.4 ^A^	24.4 ^B^	1.54	0.043
eCH_4_ (g/kg dDMI)	61.2 ^A^	58.7 ^B^	3.37	0.035
eCH_4_ (g/kg OMI)	30.3 ^A^	25.6 ^B^	1.67	0.022
eCH_4_ (g/kg dOMI)	63.6 ^A^	58.0 ^B^	4.02	0.026
Ym (% GEI)	8.3 ^A^	7.0 ^B^	0.45	0.048
eCH_4_ (g/kg milk)	72.2 ^A^	50.4 ^B^	4.10	0.001

Brach: *Brachiaria ruziziensis* hay harvested at maturity stage, Stylo: *Stylosanthes hamata* harvested at maturity stage; SEM = Standard Error of the Mean.

eCH_4_: enteric methane, BW: body weight, DMI: dry matter intake, dDMI: digestible dry matter intake, OMI: organic matter intake, dOMI: digestible organic matter intake, GEI: gross energy intake.

^A,B^ Values with different superscripts within the same row differ significantly at *p* < 0.05.

## 4 Discussion

The findings of this study demonstrate that strategic uses of locally sourced forages can simultaneously mitigate GHG emissions and enhance animal performance, an urgent priority under the converging pressures of climate change and rising protein demand in SSA. This study provides a foundation for integrating feed-based mitigation strategies into broader climate-smart livestock development frameworks.

### 4.1 Composition and nutritional quality of diets

The forages examined here are representative of species currently established and promoted across African research stations (Mutimura & Ghimire, 2021) and increasingly adopted on livestock farms (Ghimire et al., 2015; Marlon et al., 2025). According to Mutimura and Ghimire (2021), *Brachiaria* grass is among the most effective forages for improving production, ensuring year-round availability, and enhancing livestock productivity, while simultaneously addressing climate change challenges in tropical and subtropical regions. The *Brachiaria* forage tested in the current study exhibited lower nutritional quality (CP: 17.2 g/kg DM, NDF: 756.8 g/kg DM, ADF: 450.9 g/kg DM) compared with values reported by Gbenou et al. (2025) (CP: 24 g/kg DM, NDF: 773 g/kg DM, ADF: 457 g/kg DM) for hay harvested at 24 weeks, Moreira et al. (2018) (CP: 141.8 g/kg DM, NDF: 618.9 g/kg DM, ADF: 343.6 g/kg DM) as an average composition of 26 genotypes in Brazil, and Musco et al. (2016) (CP: 163.6 g/kg DM, NDF: 538 g/kg DM, ADF: 382 g/kg DM) in South Benin for the green forage. The comparatively lower quality of the Brach diet in this study is attributable to the later harvest stage of the forage.

The inclusion of *S. hamata* into the control diet significantly increased the CP content (+ 36%) without altering the NDF and ADL contents. The decline in ADF concentration observed in the Brach+Stylo diet reflects a reduced proportion of poorly digestible parietal constituents, implying improved feed degradability. This outcome is consistent with other tropical studies showing that the grass-legume associations, including *S. hamata* enhance ration nutritive value (Braga et al., 2020; Oyewole & Aderinola, 2021), by increasing soluble nitrogen availability and lowering fibre content. The role of forage legumes in improving tropical diets is well established. They provide higher-quality protein and contribute to better synchronization of energy and nitrogen in the rumen, thereby promoting microbial growth and fermentation efficiency (Quintero-Anzueta et al., 2021). The use of *S. hamata*, a species well adapted to dry tropical areas, is therefore of particular interest for suckler cow systems in SSA, where the protein deficiency in grass-based diets remains a major constraint on productivity (Abraham et al., 2023).

### 4.2 Intake, refusals and digestibility of diets

DMI obtained for the Brach diet closely matches values reported by Gbenou et al. (2025) for the same forage in zebu steers. The significant increases in DMI, OMI, and CPI observed in cows offered the Brach+Stylo diet indicate enhanced feed utilization and feeding behavior. These improvements can be attributed to greater nitrogen availability, which alleviates the primary limitation of tropical grass-based diets, their low CP content. Higher intake may also reflect improved palatability and a more balanced nutrient profile, which stimulate rumen microbial activity. Accelerated rumen fermentation may increase passage rate and voluntary intake (Ichinohe et al., 2004). Comparable responses have been reported by Pen et al. (2013), who found that ruminants consuming a *S. guianensis*-rice straw-tropical grass diet achieved greater microbial growth efficiency and feed consumption due to a more optimal energy–nitrogen balance.

Refusals accounted for approximately 28 – 29% of the DM offered and consistently showed lower CP and higher NDF content compared with the ingested fractions as reported by Gbenou et al. (2024). This pattern suggests selective feeding behavior, whereby animals preferentially consumed more digestible and nutrient-rich parts of the fractions, contributing to the increased digestibility observed in Brach+Stylo treatment.

Steers fed the Brach+Stylo diet exhibited significantly higher apparent digestibility, indicating more efficient degradation of OM and cell wall components. The higher ADF contents were negatively correlated with *in vitro* digestibility, consistent with findings by Gbenou et al. (2024). Moreover, OM digestibility was positively related to GE digestibility as reported by Gbenou et al. (2025) in tropical low-quality forages. OM digestibility in this study was lower than the value (0.63) reported by Queiroz et al. (2011), reflecting forage age. Enhanced digestibilities of OM, CP, NDF, and ADF under the Brach+Stylo diet demonstrate beneficial associative effects between grasses and legumes. These effects could be attributed to the energy-nitrogen intake synchronization, leading to more complete degradation of the plant wall and increased nutrient utilization. The negative apparent CP digestibility observed for both diets reflects endogenous fecal nitrogen, including digestive secretions, sloughed epithelial cells, mucus and microbial residues, exceeding CPI. As metabolic fecal nitrogen was not quantified, actual CP digestibility could not be estimated.

### 4.3 Impact on milk productivity of suckler cows

Cows receiving the mixed diet produced 43% more milk daily compared with those on the grass-only diet. Milk yield in the Brach treatment is comparable to the value of 1.2 kg/cow reported by Yassegoungbe et al. (2024) for non-supplemented local cows on pasture. Supplementation of the traditional tropical grass *B. ruziziensis* with the herbaceous legume *S. hamata* significantly enhanced milk production in Sudanese Fulani zebu suckler cows. This improvement reflects both the higher CP concentration (+ 36%) and the enhanced nutritive value of the legume-supplemented ration, which increased DMI (+ 16%) and DMd (+ 13%), indicating greater feed efficiency and rumen microbial activity. These results are in agreement with earlier studies demonstrating that legume supplementation improves rumen fermentation efficiency, microbial growth, and nutrient availability, thereby translating into higher milk productivity in tropical cattle (Fushai et al., 2025; Phesatcha & Wanapat, 2017).

The observed selective feeding behavior, whereby cows consumed more digestible and nutrient-dense portions of the diet, further contributed to improved nutrient assimilation and enhanced milk yield (Fushai et al., 2025; Paul et al., 2020). The enhanced synchronization of energy and nitrogen supply in the rumen, facilitated by *S. hamata*, supports microbial efficiency and improves utilization of fibrous feed components, an important determinant of milk production in suckler cows reliant on tropical forages (Hersom, 2008; Quintero-Anzueta et al., 2021). These findings underscore that incorporating locally adapted legumes into the diets of suckler cows can substantially improve milk output, as also highlighted by some previous studies (Chagunda et al., 2016; Paul et al., 2020).

### 4.4 Mitigation of enteric methane emissions

Daily eCH_4_ production (g/d) recorded in this study (averaging 98.9 g/d, 0.48 g/kg BW) are lower than values reported in bulls supplemented with crop coproducts (Somda et al., 2025), but higher than those observed in steers under similar supplementation (Gbenou et al., 2024), reflecting differences in animal category. However, variations in intake and digestibility are insufficient to elicit a statistically significant difference in daily eCH_4_ production (g/d) between treatments. These results contrast with findings by Gbenou et al. (2025) and Alvarado-Bolovich et al. (2021) who reported that higher intake and digestibility increased carbohydrate degradation and eCH_4_ production. Additional digestible nutrients from *S. hamata* may have been used more efficiently for milk synthesis rather than converted to eCH_4_, reducing eCH_4_ yield without altering absolute daily emissions. This interpretation remains speculative, as these processes were not directly measured. The 15 - 16% reduction in eCH_4_ yield (g/kg DMI or OMI) observed in the supplemented diet demonstrates that herbaceous legumes, particularly *S. hamata*, represent promising candidates for climate-smart livestock feeding. This mitigation rate exceeded the 4 - 6% reported by Somda et al. (2025) but was lower than the 20 – 23% found by Gbenou et al. (2024). Differences in intake quantity and nutrient composition likely explain these variations.

Extrapolated to one year, eCH_4_ production averaged 43.2 kg/TLU. This emission factor is lower than the 66 kg reported under IPCC Tier 1 (2019) for cows producing in average 1.4 kg milk per day in low-productivity African systems. These highlight the need to refine Tier 1 estimates by incorporating feed resource diversity and quality in the region. In contrast, the eCH₄ yield observed in this study (24 – 28 g/kg DMI) does not align with the 21.4 g/kg DMI proposed by IPCC Tier 2 (2019) for low producing cows. The average eCH_4_ yield across both treatments (26.4 g/kg DMI), also exceeds the value reported for multi-purpose cattle (23.3 g/kg DMI; IPCC, 2019), underscoring the importance of improving IPCC Tier methodologies.

The eCH_4_ intensity recorded in this study (50 – 72 g/kg milk) was lower than the 97 - 106 g/kg milk reported by Yassegoungbe et al. (2024), a difference attributable to methodologic approaches in eCH_4_ assessment. Notably, eCH_4_ yield (%GEI) and intensity (g/kg milk) decreased by 16% and 30%, respectively, in cows fed the Brach+Stylo diet compared with Brach diet. This reflects improved energy efficiency and conversion into milk production. In the context of SSA livestock systems, where productivity is typically low, supplementing *S. hamata*, a locally adapted legumes, offers a practical pathway to balance productivity gains with environmental sustainability.

### 4.5 Implications for sustainable livestock farming in sub-Saharan Africa

The incorporation of *S. hamata* into grass-based feeding systems for suckler cows in SSA represents a promising strategy for sustainable livestock farming, simultaneously enhancing productivity and reducing the environmental footprint. Although absolute daily eCH_4_ emissions did not decline significantly, both eCH_4_ yield and intensity decreased significantly, by 17% and 25%, respectively, in cows receiving the supplemented diet. These reductions indicate improved feed efficiency and energy utilization, thereby mitigating eCH_4_ emissions relative to production output. A major strength of this study is the rigorous GreenFeed® protocol. The large number of valid visits (98 per cow per diet), combined with standardized calibration and quality-control procedures, enhance confidence in the eCH_4_ measurements. Similar findings were reported by Gbenou et al. (2024), who emphasized that improving forage quality is central to increasing intake and reducing eCH_4_ emissions. This is particularly relevant in SSA, where livestock production is constrained by poor-quality feed resources and heightened sustainability challenges, especially during the dry season (Gbenou et al., 2025). Protein supplementation with *S. hamata* addresses the widespread protein deficiencies in tropical grass diets and enhances system sustainability by supporting higher productivity with lower GHG intensity. The bioactive compounds in *S. hamata*, including tannins and phenols, likely contributed to eCH_4_ reduction by modulating rumen microbial populations, thereby offering a climate-smart feeding strategy (Fushai et al., 2025). However, as these compounds were not quantified, their contribution to the observed response remains speculative.

In a region where rapid population growth and urbanisation are intensifying demand for animal-source protein WBG (2023), such feed innovations align with climate-smart livestock strategies. They improve resource use efficiency and provide a viable mitigation pathway for eCH_4_ emissions, contributing to both climate resilience and food security in SSA. Strategic integration of locally adapted forage legumes thus offers a practical and scalable solution for improving livestock productivity while reducing the environmental impact of animal agriculture in SSA.

### 4.6 Limitations of the study

A limitation of this study is that apparent digestibility was measured in steers rather than in the lactating cows used for intake and eCH_4_ measurements. Lactation is associated with higher feed intake and faster digesta passage, which may slightly reduce digestibility compared with non-lactating animals (Huhtanen et al., 2009). Thus, while the steer data provide a useful assessment of dietary quality, they may not fully represent digestibility under lactating conditions. Future studies should measure digestibility directly in lactating cows where feasible. A second limitation is that all animals received the diets in the same sequence, preventing separation of treatment and period effects. Observed responses should therefore be interpreted with caution and confirmed in randomized crossover designs.

## 5 Conclusion

This study demonstrates that supplementing tropical grass forage with the herbaceous legume *Stylosanthes hamata* significantly improved feed intake, nutrient digestibility, and milk yield in Sudanese Fulani zebu suckler cows, while reducing enteric methane yield and intensity. Absolute daily enteric methane emissions (g/d) were unchanged, suggesting that mitigation stemmed primarily from improved feed-use efficiency rather than reduced methanogenesis. These findings highlight the dual benefit of *S. hamata* supplementation in enhancing animal productivity and mitigating environmental impacts. Integrating locally adapted legumes into ruminant feeding strategies thus represents a practical and sustainable pathway to improving livestock performance and resilience in sub-Saharan Africa. Moreover, by lowering greenhouse gas intensity, this approach contributes directly to climate change mitigation and aligns with broader climate-smart livestock development strategies.

Although absolute daily enteric methane emissions (g/d) were not significantly reduced, the lower methane yield (g/kg DMI) and intensity (g/kg milk) indicate improved feed-use efficiency and reduced emissions per unit of feed consumed and milk produced. These findings support the potential of S. hamata as a locally adapted forage to enhance the environmental sustainability of suckler cow production systems in sub-Saharan Africa.

## Ethical approval

Ethical approval for this study was obtained from the CIRDES (Centre International de Recherche-Développement sur l’Élevage en zone Subhumide) Ethics Committee for Animal Experiments on 15 April 2021 (Application No 006/Mars/2021/CE-CIRDES). All procedures involving animals were conducted in strict accordance with institutional guidelines for the care and use of animals in research.

## Declaration of Generative AI and AI-assisted technologies in the writing process

During the preparation of this work the author(s) did not use any AI and AI-assisted technologies.

## Financial support statement

This study was made possible through the support of the Africa-Milk project, supported by the LEAP-Agri initiative (Grant Agreement No 727716 – LEAP-Agri) and ‘‘Carbon Sequestration and greenhouse gas emissions in (agro) Sylvopastoral Ecosystems in the sahelian CILSS States” (CaSSECS) regional project funded by the European Union (European DeSIRA programme, under grant agreement No FOOD/2019/410–169).

## Ethical Statement

All experimental procedures involving animals were conducted in accordance with institutional guidelines and regulations for the ethical treatment of animals. The study titled " Tropical legume grass forage (Stylosanthes hamata L.) mitigates enteric methane emissions and improves productivity in Fulani zebu suckler cows" was reviewed and approved by the Ethics Committee for Animal Experiments of the Centre International de Recherche-Développement sur l’Élevage en zone Subhumide (CIRDES), under application number 006/Mars/2021/CE CIRDES, dated 15 April 2021.

## CRediT authorship contribution statement

**G.X. Gbenou:** Writing – review & editing, Writing – original draft, Visualization, Formal analysis, Data curation, Conceptualization. **B.M. Orounladji:** Writing – review & editing, Writing – original draft, Formal analysis, Data curation. **B. Adama:** Writing – review & editing, Formal analysis, Data curation, Conceptualization. **F. Sanou:** Writing – review & editing, Investigation, Conceptualization. **D. Bastianelli:** Writing – review & editing, Formal analysis, Conceptualization. **O. Sib:** Writing – review & editing, Supervision, Funding acquisition, Conceptualization. **S. Sanogo:** Writing – review & editing, Supervision, Conceptualization. **L. Bonnal:** Writing – review & editing, Methodology, Formal analysis. **E. Vall:** Writing – review & editing, Funding acquisition, Conceptualization. **L.H. Dossa:** Writing – review & editing, Writing – original draft, Supervision, Conceptualization. **M.H. Assouma:** Writing – review & editing, Writing – original draft, Supervision, Methodology, Funding acquisition, Formal analysis, Data curation, Conceptualization.

## Declaration of competing interest

The authors whose names are listed immediately below certify that they have no conflict, NO affiliations with or involvement in any organization or entity with any financial interest (such as honoraria; educational grants; participation in speakers’ bureaus; membership, employment, consultancies, stock ownership, or other equity interest; and expert testimony or patent-licensing arrangements), or non-financial interest (such as personal or professional relationships, affiliations, knowledge or beliefs) in the subject matter or materials discussed in this manuscript. The manuscript represents valid work; neither this manuscript nor one with substantially similar content under our authorship has been published or is being considered for publication elsewhere (except as described in the manuscript submission); and copies of any closely related manuscripts are enclosed in the manuscript submission.

G. X. Gbenou, B. M. Orounladji, B. Adama, F. Sanou, D. Bastianelli, O. Sib, S. Sanogo, L. Bonnal, E. Vall, L. H. Dossa, M. H. Assouma.

## Data Availability

The datasets generated and analyzed during the current study are not publicly available but are available from the corresponding author upon reasonable request.
